# Supplementary material for: Drug-Tolerant Idling Melanoma Cells Exhibit Theory-Predicted Metabolic Low-Low Phenotype
Source: Front Oncol. 2020 Aug 14;10:1426. doi: 10.3389/fonc.2020.01426 (PMC7457027; doi:10.3389/fonc.2020.01426)
Supplement: Supplementary file 1 [file Data_Sheet_1.docx]

**SUPPLEMENTARY INFORMATION FOR:**

**Drug-tolerant idling melanoma cells exhibit theory-predicted metabolic low-low phenotype**

**Authors/Affiliations:**

Dongya Jia^1,^*, B. Bishal Paudel^2,3,4,^*, Corey E. Hayford^2,3,5^, Keisha N. Hardeman^2,3^, Herbert Levine^1,6,7,#^, José N. Onuchic^1,8,9,10,#^ and Vito Quaranta^2,3,#^

^1^Center for Theoretical Biological Physics, Rice University, Houston, TX 77005;

^2^Department of Biochemistry, Vanderbilt University, Nashville, TN 37232;

^3^Quantitative Systems Biology Center, Vanderbilt University, Nashville, TN 37232;

^4^Department of Biomedical Engineering, University of Virginia, Charlottesville, VA 22908;

^5^Chemical and Physical Biology Graduate Program, Vanderbilt University, Nashville, TN 37232;

^6^Department of Bioengineering, Northeastern University, Boston, MA 02115, USA

^7^Department of Physics, Northeastern University, Boston, MA 02115, USA

^8^Department of Biosciences, Rice University, Houston, TX 77005;

^9^Department of Physics and Astronomy, Rice University, Houston, TX 77005;

^10^Department of Chemistry, Rice University, Houston, TX 77005;

*These authors contributed equally.

^#^These authors contributed equally.

Address Correspondence to:

Vito Quaranta ([vito.quaranta@vanderbilt.edu](mailto:vito.quaranta@vanderbilt.edu))

Herbert Levine ([h.levine@northeastern.edu](mailto:h.levine@northeastern.edu))

Jose Onuchic ([jonuchic@rice.edu](mailto:jonuchic@rice.edu))

**Table of Contents:**

[**Supplementary Note:**](#_gjdgxs)

**1.** [**Mathematical Modeling of Cancer Metabolism**](#_30j0zll)

**2.** [**Estimation of Metabolic Parameters from Seahorse Metabolic Assay**](#_3znysh7)

**3.** [**Mathematical Model for Differential Cell Metabolism and Cell Population Dynamics**](#_2et92p0)

**Supplementary Tables:**

[**Table S1. The values of parameters used for Figures 1A-C**](#_1t3h5sf)

[**Table S2. The values of parameters used for Figures 1D-E***](#_4d34og8)

[**Table S3. AMPK and HIF-1 signature genes**](#_2s8eyo1)

[**Table S4. Metabolic pathway signature genes**](#_17dp8vu)

[**Table S5. Gene Expression Data for Drug Treatment Time Course Subclones**](#_3rdcrjn)

**Table S6. Treatment information of the patient samples obtained from GEO with the series ID GSE75299**

[**Table S7. Genes used for Principal Component Analysis (PCA) in Figure 2G**](#_3dy6vkm)

[**Supplementary Data:**](#_26in1rg)

[**Supplementary Figures:**](#_1ksv4uv)

[**Supplementary References:**](#_2jxsxqh)

**Supplementary Note:**

## **Mathematical Modeling of Cancer Metabolism**

To capture the coupling of gene regulation and metabolic pathways, we used our mathematical model of cancer metabolism devised in our previous work [(1)](https://paperpile.com/c/L3NbwQ/pBq00). To simulate the temporal dynamics of pAMPK ($A$), HIF-1 ($H$), mtROS ($R_{mt}$), noxROS ($R_{nox}$) and total level of ROS ($R_{T}$), the following equations are devised.

$$\dot{A}= g_{A}H^{s+}\left( R_{T}, {R_{T,A}^{0},\lambda}_{R_{T}, A},n_{R_{T}, A} \right)H^{s-}\left( H,H_{A}^{0}, \lambda_{H, A},n_{H, A} \right)H^{s-}\left( X_{ATP}, {X_{ATP,A}^{0},\lambda}_{X_{ATP},A},n_{X_{ATP},A} \right)- k_{A}A (eq. 1)$$

$$\dot{H}= g_{H}H^{s-}\left( A,A_{H}^{0}, \lambda_{A,H},n_{A,H} \right)-k_{H}\cdot{H\cdot H}^{s-}\left( G_{2},G_{2, H}^{0} ,\lambda_{G_{2},H},n_{G_{2},H} \right)H^{s-}\left( R_{T}, {R_{T,H}^{0},\lambda}_{R_{T}, H},n_{R_{T}, H} \right) (eq. 2)$$

$$\dot{R}_{mt}= g_{R_{mt}}\left( \gamma_{G_{1}}G_{1}+\gamma_{F}F \right)-k_{R_{mt}}R_{mt}H^{s+}\left( A,A_{R_{mt}}^{0}, \lambda_{A,R_{mt}},n_{A,R_{mt}} \right) (eq. 3)$$

$$\dot{R}_{nox}= g_{R_{nox}}C_{R_{nox}}^{comp}\left( g_{0},H,{g_{H, R_{nox}}, H}_{R_{nox}}^{0},n_{{H, R}_{nox}},A{,g_{A, R_{nox}}, A}_{R_{nox}}^{0},n_{{A, R}_{nox}} \right)-k_{R_{nox}}R_{nox} (eq. 4)$$

$$R_{T}=R_{mt}+R_{nox} (eq. 5)$$

As the chemical reactions in the metabolic processes are much faster than the genetic regulation, we assume that the metabolic pathways are in the equilibrium state at certain levels of the regulatory proteins, pAMPK and HIF-1. To capture the dynamics of the metabolic fluxes, the following equations are devised to simulate the glucose uptake rate ($G_{0}$), the glucose consumption rate ($G$), the utilization rate of acetyl-CoA for mitochondrial respiration ($C_{0}$), the production rate of acetyl-CoA ($C$), the glucose oxidation rate $(G_{1})$, the glycolysis rate ($G_{2}$), the fatty acid oxidation rate $(F)$, the ATP production rate of glucose oxidation ($G_{1,ATP}$), the ATP production rate of glycolysis ($G_{2,ATP}$), the ATP production rate of fatty acid oxidation ($F_{ATP}$), the total ATP production rate ($X_{ATP}$).

$$G_{0}=g_{H,G_{0}}H^{s+}\left( H,H_{G_{0}}^{0}, \lambda_{H, G_{0}},n_{H, G_{0}} \right)+g_{A, G_{0}}H^{s+}\left( A,A_{G_{0}}^{0}, \lambda_{A, G_{0}},n_{A, G_{0}} \right) (eq. 6)$$

$$G=G_{1}+G_{2} (eq.7)$$

$$C_{0}=g_{A, C_{0}}H^{s+}\left( A,A_{C_{0}}^{0}, \lambda_{A, C_{0}},n_{A, C_{0}} \right) (eq.8)$$

$$C=2*G_{1}+9*F (eq.9)$$

$$G_{1}= g_{G_{1}}H^{s-}\left( G,G_{0}, \lambda_{G, G_{1}},n_{G, G_{1}} \right)H^{s-}\left( C,C_{0}, \lambda_{C, G_{1}},n_{C, G_{1}} \right) (eq. 10)$$

$$G_{2}= g_{G_{2}}H^{s-}\left( G,G_{0}, \lambda_{G, G_{2}},n_{G, G_{2}} \right)H^{s+}\left( H,H_{G_{2}}^{0}, \lambda_{H, G_{2}},n_{H, G_{2}} \right) (eq.11)$$

$$F= g_{f}H^{s-}\left( C,C_{0}, \lambda_{C, F},n_{C, F} \right)H^{s+}\left( A,A_{F}^{0}, \lambda_{A, F},n_{A,F} \right) (eq.12)$$

$$G_{1,ATP}=29*G_{1} (eq.13)$$

$$G_{2,ATP}=2*G_{2} (eq.14)$$

$$F_{ATP}=106*F (eq.15)$$

$$X_{ATP}=G_{1,ATP} + G_{2,ATP} + F_{ATP} (eq.16)$$

All the equation files and description are the same as shown in the supplementary information in our previous work (1). Some key biological features that were considered in the model are as follows. The total level of ROS is equal to the sum of the mtROS level and the noxROS level, as shown in $(eq. 5)$. The total glucose consumption rate is equal to the sum of glucose consumption rate of glucose oxidation and glycolysis, as shown in $(eq. 7)$. The total production rate of Acetyl-CoA that fuels the TCA cycle for ATP production is equal to the sum of Acetyl-CoA production rate of glucose oxidation and fatty acid oxidation, as shown in $(eq.9)$. The total production rate of ATP is equal to the sum of the ATP production rate of glucose oxidation, the production rate of ATP of glycolysis and ATP production rate of fatty acid oxidation, as shown in $(eq.16)$. The explanation of each parameter can be found in ***Table S2***. The parameters of the metabolic model were estimated per experimental evidence and not fitted to any data. Additional details can be found in (1).

## **Estimation of Metabolic Parameters from Seahorse Metabolic Assay**

From metabolic assay data, we extracted eight metabolic parameters (four from each Mito Stress and Glyco Stress test) based on the schematic and information provided by Agilent ([https://www.agilent.com](https://www.agilent.com/)) and as previously described [(2)](https://paperpile.com/c/L3NbwQ/238dq).


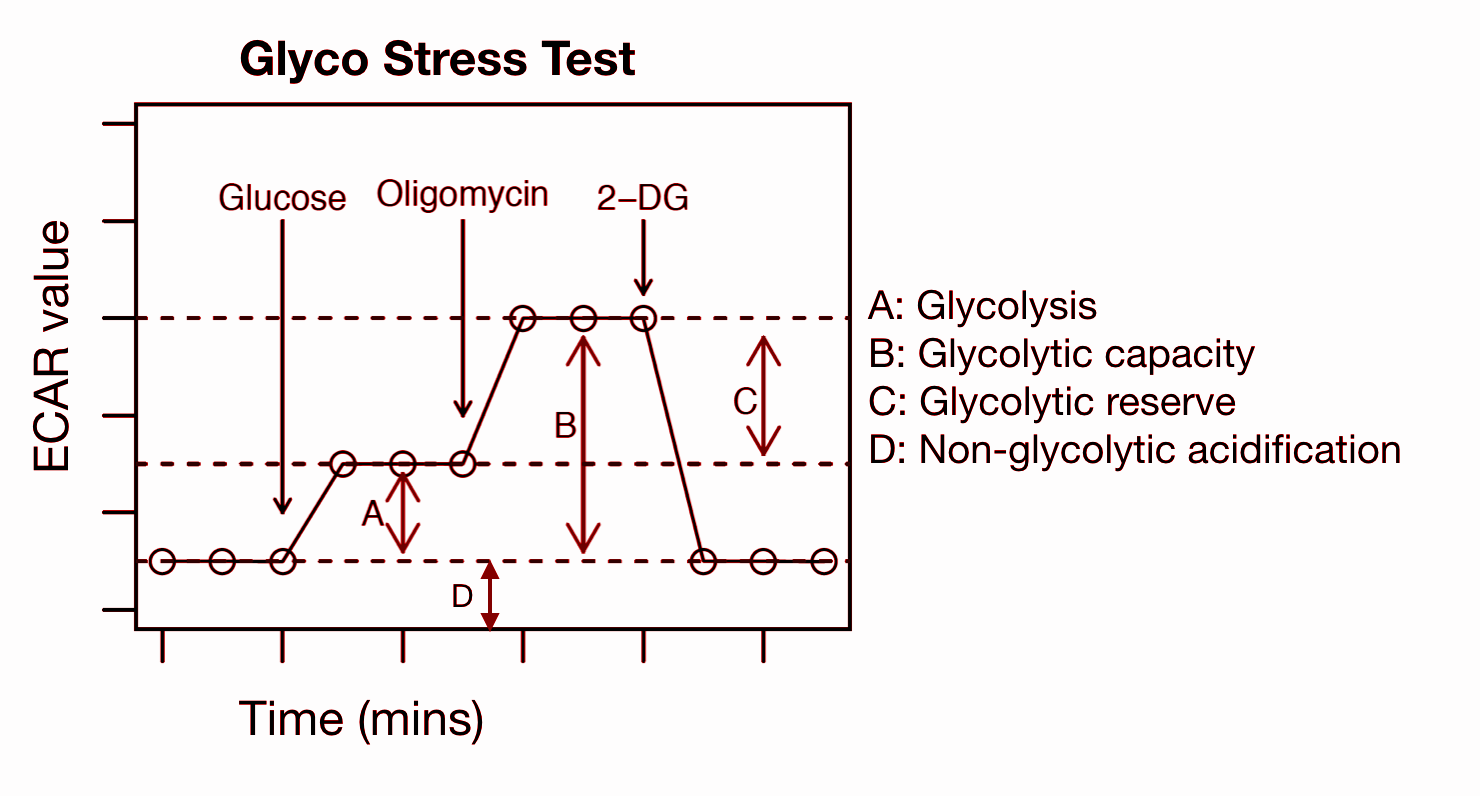


Schematic 1A: Glyco Stress Test according to Agilent Instructions.


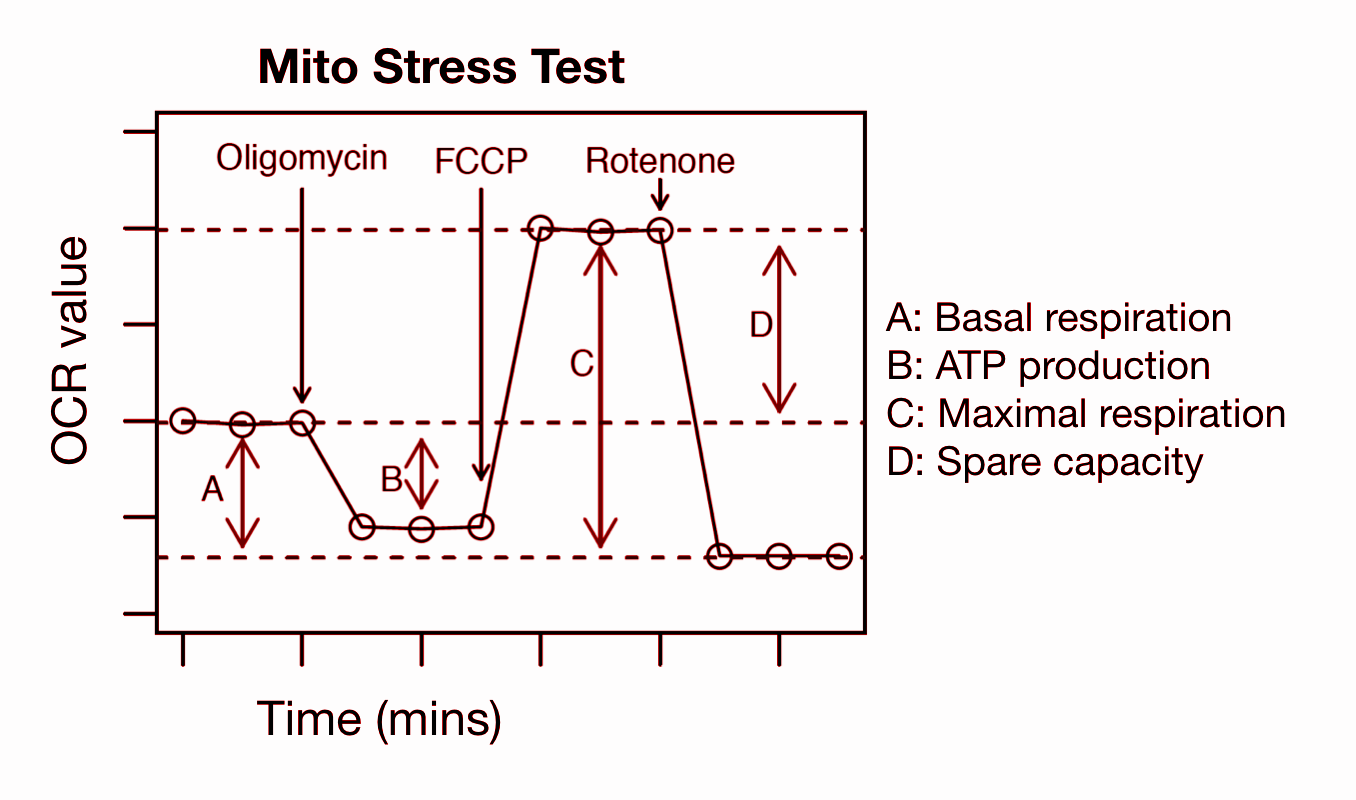


Schematic 1B: Mito Stress Test according to Agilent Instructions.

## **Mathematical Model for Differential Cell Metabolism and Cell Population Dynamics**

We postulate that the growth dynamics of cancer cells is a function of their oxidative phosphorylation (OXPHOS) capacity, $f\left( oxphos \right)$ and their glycolytic capacity $g\left( glycolysis \right)$.

$proliferation=f\left( oxphos \right)+g\left( glycolysis \right) (eq.17)$

Linking the full metabolic characteristics with proliferation behaviors of cancer cells is beyond the scope of this manuscript, and will require a major undertaking. In particular, it would require accurately measuring energy production via OXPHOS and/or glycolysis, and quantifying their contribution towards the cell growth and cell death. We believe that the cellular proliferation is linked to the bioenergetic state of cells via some non-linear function as in $(eq.17)$. For simplicity, we considered a phenomenological model of cell proliferation dynamics in this manuscript. We assume that cancer cells can exist in three distinct metabolic states: OXPHOS^high^Glycolysis^high^ (High/High or H), OXPHOS^high^Glycolysis^low^ or OXPHOS^low^Glycolysis^high^ (High/Low or Low/High or I (Intermediate)), and  OXPHOS^low^Glycolysis^low^ (Low/Low or L). Cells in each state can divide, die, or transition into other states, as described in [(3)](https://paperpile.com/c/L3NbwQ/eOec). The set of ordinary differential equations describing the temporal dynamics of each state are as follows:

$\frac{dn_{H}}{dt}={(\mu}_{H}-k_{hi}-k_{hl})*n_{H}+ k_{ih}*n_{I}+ k_{lh}*n_{L} (eq.18)$

$\frac{dn_{I}}{dt}={(\mu}_{I}-k_{ih}-k_{il})*n_{I}+ k_{hi}*n_{H}+ k_{li}*n_{L} (eq. 19)$

$\frac{dn_{L}}{dt}={(\mu}_{L}-k_{li}-k_{lh})*n_{L}+ k_{il}*n_{I}+ k_{hl}*n_{H} (eq. 20)$

where $n_{H}$, $n_{I}$, and $n_{L}$ represent the numbers of cells in the metabolic states of *H*, *I* and *L* respectively;$\mu_{H}, \mu_{I}$ and $\mu_{L}$ represent the net-proliferation rates for metabolic states *H*, *I* and *L* respectively. $k_{hi}$ and $k_{ih}$ represent the transition rate constants between states *H* and *I*; $k_{hl}$ and $k_{lh}$ represent the transition rate constants between states *H* and *L*; $k_{il} and k_{li}$represent the transition rate constants between states *I* and *L*. Based on recent studies that suggest BRAF inhibitors inhibit glycolytic pathway, we assumed that:

1. Cells in *H* state will have a net-positive proliferation rate ($\mu_{H}=0.02 doublings/h$)
2. Cells in *L* state will have a net-zero proliferation rates ($\mu_{L}=0.00 doublings/h)$
3. Cells in *I* state will have a net-negative proliferation rate ($\mu_{I}= -0.04 doublings/h)$

The remaining six transition rate parameters between metabolic states, $k_{hi}$, $k_{ih}$, $k_{hl}$, $k_{lh},$ $k_{il}$ and $k_{li}$ were set to be between $0\leq k\leq0.06 h^{-1}$ and calibrated to the experimental data of different subclones. The upper limit $0.06 h^{-1}$ on the transition rate constants was set to be less than the drug-free proliferation rates of the cancer cells [(4)](https://paperpile.com/c/L3NbwQ/XSZx). The initial cell proportions, $I_{0} and L_{0}$were set to vary between 0 and 1 and $H_{0}$was calculated as ${1-I}_{0}-L_{0}$. The model was encoded in R ([www.r-project.org](http://www.r-project.org)), and ordinary differential equation simulations were performed using the ‘*ode*’ function of the R package “*deSolve*” [(5)](https://paperpile.com/c/L3NbwQ/X3Yr). Parameter calibration was performed using Monte Carlo Markov Chain (MCMC) sampling (~50,000 iterations) using the ‘*modMCMC*’ function of the R package ‘*FME*’ [(6)](https://paperpile.com/c/L3NbwQ/LyZi). Goodness of fit was quantified using the cost function:

$Cost=\sum_{i=1}^{n} \frac{\left( M_{i}-O_{i} \right)^{2}}{\sigma_{i}} (eq. 21)$

where *n* is the number of measured time points and $M_{i}$, $O_{i}$, and $\sigma_{i}$ are the model prediction, experimentally observed value, and standard experimental error (automatically determined by *modMCMC*) at time point $i$, respectively. For each subclone, the model was calibrated against the experimental time course for the respective subclones, SC01, SC07, and SC10. Predictions for the dynamics of each subclone were then made by selecting 1000 random parameter sets from the last 50% of the MCMC iterations (accounting for burn-in) in the MCMC-generated parameter ensemble. In all cases, we plot the simulated time courses as envelopes within one standard deviation of the mean based on 1000 random samples of the MCMC-generated parameter ensemble.

**Supplementary Tables:**

## **Table S1. The values of parameters used for Figures 1A-C**

| **Parameters** | **Value** | **Unit** | **Description** |
| --- | --- | --- | --- |
| $g_{R_{mt}}$ | 35 | $nmol\cdot L^{-1}\cdot h^{-1}$ | Production rate of mtROS |
| $\gamma_{G_{1}}$ | 1 | - | mtROS production in glucose oxidation |
| $\gamma_{F}$ | 9/2 | - | mtROS production in fatty acid oxidation |
| $k_{R_{mt}}$ | 5 | ${min}^{-1}$ | Degradation rate of mtROS |
| $A_{R_{mt}}^{0}$ | 350 | $nmol\cdot L^{-1}$ | Threshold of mtROS inhibition by AMPK |
| $\lambda_{A,R_{mt}}$ | 2 | - | Fold change of mtROS inhibition by AMPK |
| $n_{A,R_{mt}}$ | 2 | - | Hill coefficient |
| $g_{R_{nox}}$ | 40 | $\mu mol\cdot L^{-1}\cdot{min}^{-1}$ | Production rate of noxROS |
| $g_{0}$ | 1 | - | Basal noxROS |
| $g_{H, R_{nox}}$ | 5 | - | Fold-change of noxROS activation by HIF-1 |
| $H_{R_{nox}}^{0}$ | 250 | $nmol\cdot L^{-1}$ | Threshold of noxROS activation by HIF-1 |
| $n_{{H, R}_{nox}}$ | 2 | - | Hill coefficient |
| $g_{A, R_{nox}}$ | 0.2 | - | Fold change of noxROS inhibition by AMPK |
| $A_{R_{nox}}^{0}$ | 150 | $nmol\cdot L^{-1}$ | Threshold of noxROS inhibition by AMPK |
| $n_{{A, R}_{nox}}$ | 2 | - | Hill coefficient |
| $k_{R_{nox}}$ | 5 | ${min}^{-1}$ | Degradation rate of noxROS |
| $g_{A}$ | 40 | $nmol\cdot L^{-1}\cdot h^{-1}$ | Production rate of AMPK |
| $R_{T,A}^{0}$ | 250 | $\mu mol\cdot L^{-1}$ | Threshold of AMPK activation by ROS |
| $\lambda_{R_{T}, A}$ | 8 | - | Fold change of AMPK activation by ROS |
| $n_{R_{T}, A}$ | 4 | - | Hill coefficient |
| $H_{A}^{0}$ | 150 | $nmol\cdot L^{-1}$ | Threshold of AMPK inhibition by HIF-1 |
| $\lambda_{H, A}$ | 0.1 | - | Fold-change of AMPK inhibition by HIF-1 |
| $n_{H, A}$ | 1 | - | Hill coefficient |
| $\lambda_{X_{ATP},A}$ | 0.25 | - | Fold-change of AMPK inhibition by ATP |
| $X_{ATP,A}^{0}$ | 2000 | $\mu mol\cdot L^{-1}\cdot s^{-1}$ | Threshold of AMPK inhibition by ATP |
| $n_{X_{ATP},A}$ | 2 | - | Hill coefficient |
| $k_{A}$ | 0.2 | $h^{-1}$ | Degradation rate of AMPK |
| $g_{H}$ | 15 |  | Production rate of HIF-1 |
| $A_{H}^{0}$ | 150 | $nmol\cdot L^{-1}$ | Threshold of HIF-1 inhibition by AMPK |
| $\lambda_{A,H}$ | 0.1 | - | Fold-change of HIF-1 inhibition by AMPK |
| $n_{A,H}$ | 1 | - | Hill coefficient |
| $k_{H}$ | 0.25 | $h^{-1}$ | Degradation rate of HIF-1 |
| $G_{2, H}^{0}$ | 250 | $\mu mol\cdot L^{-1}\cdot s^{-1}$ | Threshold of HIF-1 activation by glycolysis |
| $\lambda_{G_{2},H}$ | 0.1 | - | Fold-change of HIF-1 activation by glycolysis |
| $n_{G_{2},H}$ | 4 | - | Hill coefficient |
| $\lambda_{R_{T}, H}$ | 0.2 | - | Fold-change of HIF-1 stabilization by ROS |
| $R_{T,H}^{0}$ | 40 | $\mu mol\cdot L^{-1}$ | Threshold of HIF-1 stabilization by ROS |
| $n_{R_{T}, H}$ | 4 | - | Hill coefficient |
| $g_{H,G_{0}}$ | 20 | $\mu mol\cdot L^{-1}\cdot s^{-1}$ | Basal glucose uptake rate |
| $H_{G_{0}}^{0}$ | 150 | $nmol\cdot L^{-1}$ | Threshold of glucose uptake regulated by HIF-1 |
| $\lambda_{H, G_{0}}$ | 6 | - | Fold-change of glucose uptake regulated by HIF-1 |
| $n_{H, G_{0}}$ | 4 | - | Hill coefficient |
| $g_{A, G_{0}}$ | 20 | $\mu mol\cdot L^{-1}\cdot s^{-1}$ | Basal glucose uptake rate |
| $A_{G_{0}}^{0}$ | 200 | $nmol\cdot L^{-1}$ | Threshold of glucose uptake regulated by AMPK |
| $\lambda_{A, G_{0}}$ | 4 | - | Fold-change of glucose uptake regulated by AMPK |
| $n_{A, G_{0}}$ | 2 | - | Hill coefficient |
| $g_{A, C_{0}}$ | 15 | $\mu mol\cdot L^{-1}\cdot s^{-1}$ | Basal utilization rate of acetyl-CoA for OXPHOS |
| $A_{C_{0}}^{0}$ | 250 | $nmol\cdot L^{-1}$ | Threshold of acetyl-CoA utilization regulated by AMPK |
| $\lambda_{A, C_{0}}$ | 8 | - | Fold-change of acetyl-CoA utilization regulated by AMPK |
| $n_{A, C_{0}}$ | 4 | - | Hill coefficient |
| $g_{G_{1}}$ | 100 | $\mu mol\cdot L^{-1}\cdot s^{-1}$ | Basal glucose oxidation rate |
| $\lambda_{G, G_{1}}$ | 0.1 | - | Restriction of glucose oxidation rate by glucose uptake rate |
| $n_{G, G_{1}}$ | 2 | - | Hill coefficient |
| $\lambda_{C, G_{1}}$ | 0.1 | - | Restriction of glucose oxidation by acetyl-CoA utilization rate |
| $n_{C, G_{1}}$ | 4 | - | Hill coefficient |
| $g_{G_{2}}$ | 150 | $\mu mol\cdot L^{-1}\cdot s^{-1}$ | Basal glycolysis rate |
| $\lambda_{G, G_{2}}$ | 0.1 | - | Restriction of glycolysis rate by glucose uptake rate |
| $n_{G, G_{2}}$ | 2 | - | Hill coefficient |
| $H_{G_{2}}^{0}$ | 200 | $nmol\cdot L^{-1}$ | Threshold of glycolysis upregulation by HIF-1 |
| $\lambda_{H, G_{2}}$ | 8 | - | Fold-change of glycolysis upregulation by HIF-1 |
| $n_{H, G_{2}}$ | 4 | - | Hill coefficient |
| $g_{f}$ | 2 | $\mu mol\cdot L^{-1}\cdot s^{-1}$ | Basal FAO rate |
| $\lambda_{C, F}$ | 0.1 | - | Restriction of FAO rate by acetyl-CoA utilization rate |
| $n_{C, F}$ | 2 | - | Hill coefficient |
| $A_{F}^{0}$ | 200 | $nmol\cdot L^{-1}$ | Threshold of FAO upregulation by AMPK |
| $\lambda_{A, F}$ | 6 | - | Fold-change of FAO upregulation by AMPK |
| $n_{A,F}$ | 4 | - | Hill coefficient |

## **Table S2. The values of parameters used for Figures 1D-E***

| **Parameters** | **Value** | **Unit** | **Description** |
| --- | --- | --- | --- |
| **High mtROS, high HIF-1** | | | |
| $g_{R_{mt}}$ | 45 | $nmol\cdot L^{-1}\cdot h^{-1}$ | Production rate of mtROS |
| $k_{H}$ | 0.25 | $h^{-1}$ | Degradation rate of HIF-1 |
| **High mtROS, low HIF-1** | | | |
| $g_{R_{mt}}$ | 45 | $nmol\cdot L^{-1}\cdot h^{-1}$ | Production rate of mtROS |
| $k_{H}$ | 0.45 | $h^{-1}$ | Degradation rate of HIF-1 |
| **Low mtROS, high HIF-1** | | | |
| $g_{R_{mt}}$ | 30 | $nmol\cdot L^{-1}\cdot h^{-1}$ | Production rate of mtROS |
| $k_{H}$ | 0.25 | $h^{-1}$ | Degradation rate of HIF-1 |
| **Low mtROS, low HIF-1** | | | |
| $g_{R_{mt}}$ | 30 | $nmol\cdot L^{-1}\cdot h^{-1}$ | Production rate of mtROS |
| $k_{H}$ | 0.45 | $h^{-1}$ | Degradation rate of HIF-1 |

*The values of all other parameters are the same as listed in **Table S1**.

## **Table S3. AMPK and HIF-1 signature genes**

| **AMPK signature genes** | **HIF-1 signature genes** |
| --- | --- |
| ACADL | ALDH4A1 |
| ACADM | ALDOA |
| ACOX1 | BHLHE40 |
| ACSL1 | BNIP3 |
| ACSL5 | CA9 |
| ANGPTL4 | CCNB1 |
| APOC3 | DDIT4 |
| APOE | EGLN3 |
| ATF4 | EPRS |
| BAX | ETS1 |
| CAT | IVNS1ABP |
| CCND2 | KDM3A |
| CPT1A | MECOM |
| CPT2 | MXD1 |
| CYP27A1 | PGK1 |
| CYP4A11 | SERPINE1 |
| CYP7A1 | SSRP1 |
| DNMT1 | STC2 |
| EHHADH | TFRC |
| FOXA2 | TGFB3 |
| G6PC | TMEFF1 |
| G6PC3 | TMEM45A |
| GADD45A | VEGFA |
| GADD45G |  |
| HNF4A |  |
| MMP9 |  |
| ONECUT2 |  |
| PCK1 |  |
| PCK2 |  |
| PDK4 |  |
| PRMT1 |  |
| RUVBL1 |  |
| TOB1 |  |

## **Table S4. Metabolic pathway signature genes**

| **TCA** | **FAO** | **Glycolysis** |
| --- | --- | --- |
| ACO2 | ECHS1 | HK1 |
| IDH1 | HADH | GPI |
| OGDH | ACAA1 | PFKM |
| SDHA | ACAA2 | TPI1 |
| SDHC | CD36 | GAPDH |
| FH | SLC25A20 | PGAM2 |
| MDH1 | IVD | ENO1 |
| CS | ACADS | PKM |
| PC | GCDH |  |
| PDHA1 | ACADVL |  |
|  | ACADSB |  |
|  | ACAD8 |  |
|  | ACAD9 |  |
|  | ACAD10 |  |

##

## **Table S5. Gene Expression Data for Drug Treatment Time Course Subclones**

The RNASeq feature counts for three subclones, and their drug-treated samples can be found at: (<https://github.com/paudelbb/Metabolic_Low_Low_Data>).

**Table S6. Treatment information of the patient samples obtained from GEO with the series ID GSE75299**

| Patient # | Condition | Description |
| --- | --- | --- |
| 1 | baseline | pre MAPKi treatment |
|  | D85 | during BRAFi treatment, day 85 |
| 3 | baseline | pre MAPKi treatment |
|  | D22 | during BRAFi and MEKi treatment, day 22, |
| 4 | baseline | pre MAPKi treatment |
|  | D15 | during BRAFi and MEKi treatment, day 15 |
|  | RD261 | during BRAFi and MEKi treatment, day 261 |
| 6 | Baseline | pre MAPKi treatment |
|  | D6 | during BRAFi treatment, day 6 |
|  | D12 | during BRAFi and MEKi treatment, day 12 |
|  | D15 | during BRAFi and MEKi treatment, day 15 (using a combination of two different BRAFi and MEKi) |
| 7 | baseline | pre MAPKi treatment, |
|  | D15A | during MEKi treatment, day 15, 1st biopsy |
|  | D15B | during MEKi treatment, day 15, 2nd biopsy |
|  | D15C | during MEKi treatment, day 15, 3rd biopsy |
| 8 | baseline | pre MAPKi treatment |
|  | D22 | during BRAFi and MEKi treatment, day 22 |

## **Table S7. Genes used for Principal Component Analysis (PCA) in Figure 2G**

The following gene lists were used for gene expression Principal Component Analysis (PCA) performed in **Figure 2G** (<https://github.com/paudelbb/Metabolic_Low_Low_Data>). Briefly, expression across the samples were selected for the genes in both Hallmark Glycolysis and Hallmark Oxidative Phosphorylation. Lists were obtained from Molecular Signatures Database (MSigDB) (<http://software.broadinstitute.org/gsea/msigdb/index.jsp>).

# **Supplementary Data:**

For additional data and codes used in the preparation of this manuscript, please refer to:

(<https://github.com/paudelbb/Metabolic_Low_Low_Data>).

# **Supplementary Figures:**


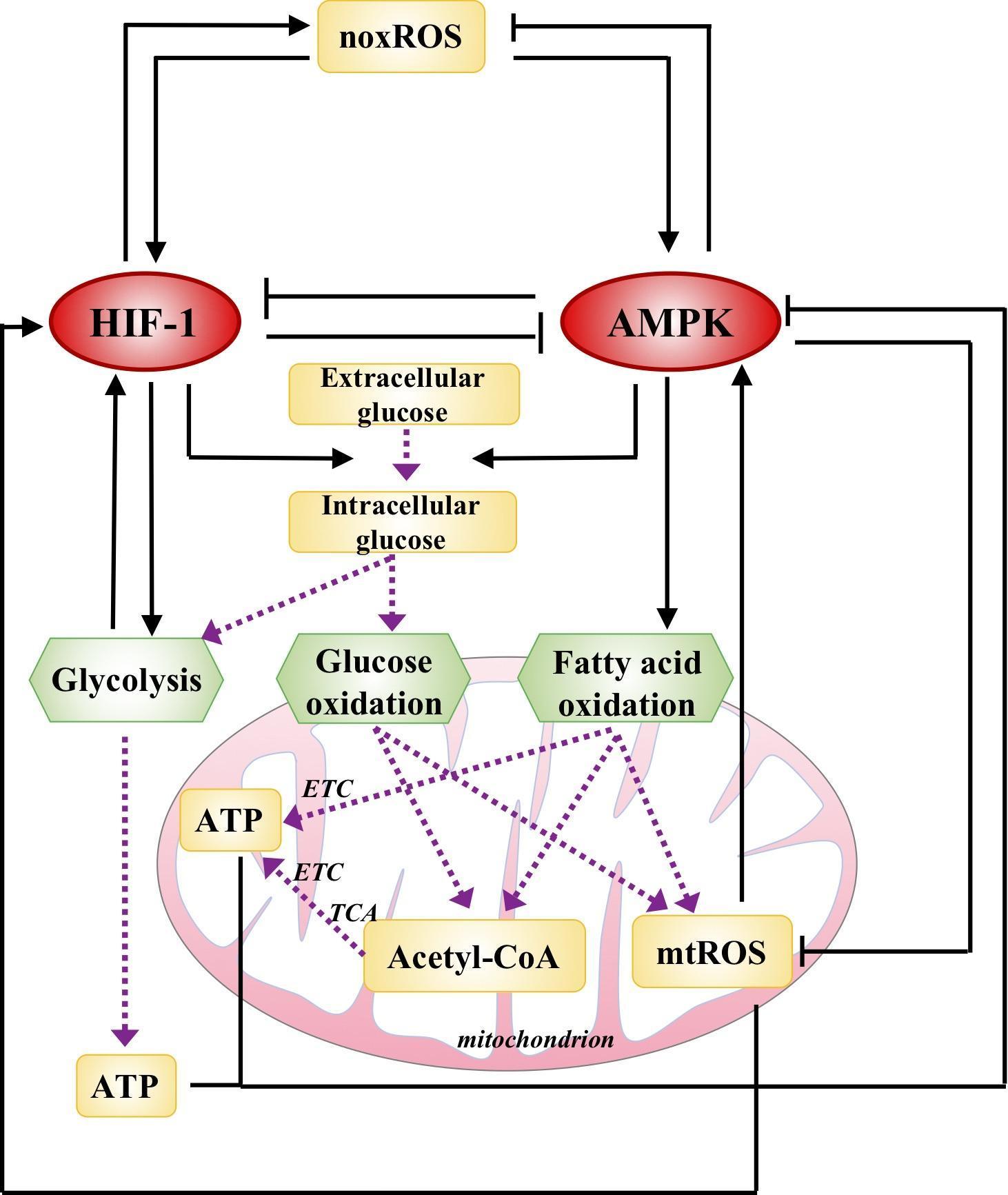


**Supplementary Figure1 (FigS1):** A minimal kinetic model that couples the regulatory circuit AMPK:HIF-1:ROS with three major metabolic pathways - glycolysis, glucose oxidation and fatty acid oxidation. The black solid arrows represent regulatory links. The purple dotted arrows represent metabolic flux. The red ovals represent two master gene regulators of metabolism - HIF-1 and AMPK. The hexagons represent three major metabolic pathways. The rectangles represent metabolites. Both AMPK and HIF-1 can regulate the uptake of glucose. The intracellular glucose can be used by glycolysis and glucose oxidation. Both glucose oxidation and fatty acid oxidation can generate acetyl-CoA to fuel the TCA for ATP production, and generate mtROS which in turn regulates AMPK and HIF-1. All three metabolic pathways produce ATP which can regulate AMPK activity. This figure is adapted from [(1)](https://paperpile.com/c/L3NbwQ/pBq00).


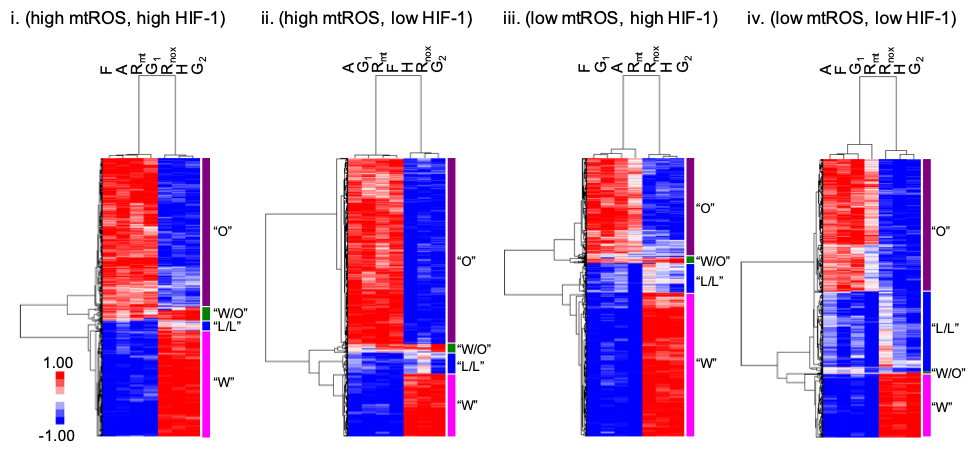


**Supplementary Figure2 (FigS2):** Hierarchical clustering analysis of the stable-state solutions from 500 sets of parameters representing the scenarios i. (high mtROS, high HIF-1), ii. (high mtROS, low HIF-1), iii. (low mtROS, high HIF-1) and iv. (low mtROS, low HIF-1). Each row represents one solution and each column represents the level of a regulatory protein, metabolite or the rate of one metabolic pathway. “F” represents FAO rate. “A” represents the level of phosphorylated AMPK. “R_mt_” represents mtROS level. “G_1_” represents glucose oxidation rate. “R_nox_” represents noxROS level. “H” represents HIF-1 level. “G_2_” represents glycolysis rate. The metabolic phenotypes corresponding to different clusters are labelled. “O” represents an OXPHOS phenotype. “W/O” represents a hybrid metabolic phenotype. “L/L” represents a metabolically low-low phenotype. “W” represents a glycolysis phenotype. Consistent with the results shown in Figure 1D-E, the fraction of “L/L” phenotype increases from left to right, as the level of mtROS and HIF-1 decreases.


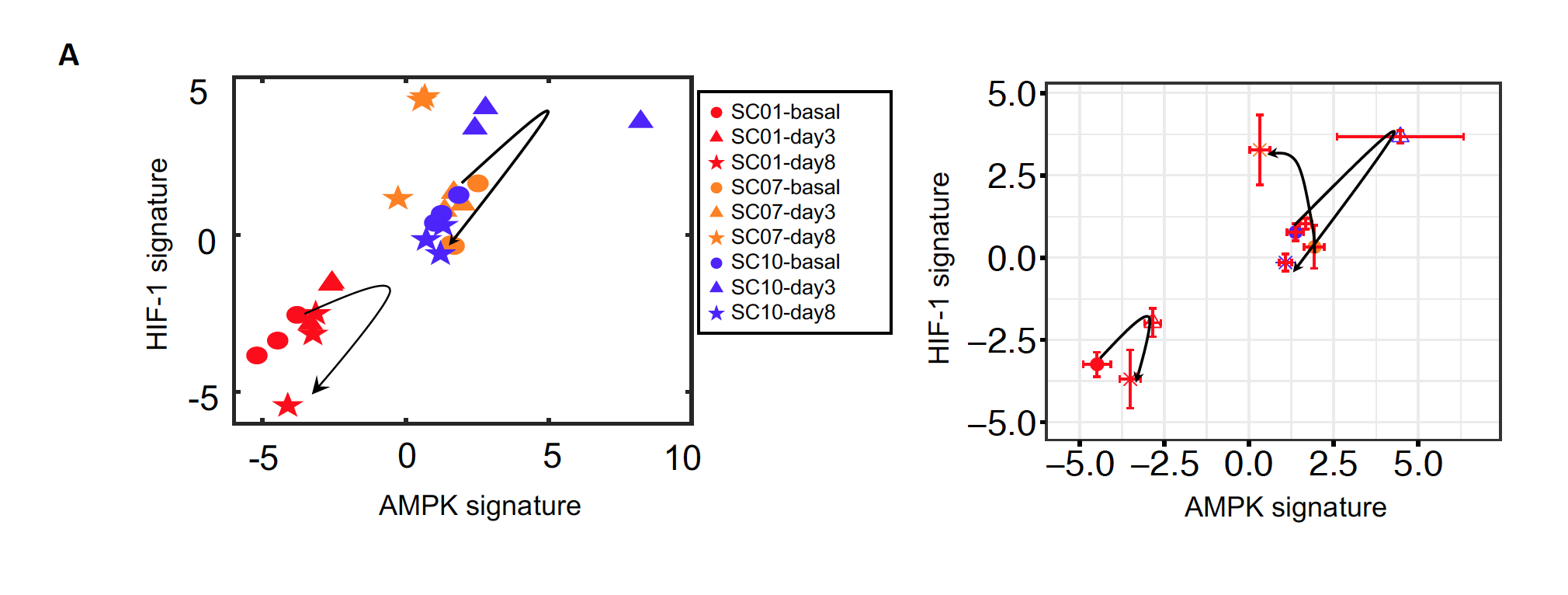


**Supplementary Figure 3 (FigS3)**: The AMPK and HIF-1 signatures of SC01, SC07 and SC10 before and during the treatment of 8uM PLX4720 at day 3 and day 8. Left panel: the AMPK and HIF-1 signatures of each replica for the indicated time points of each subclone; right panel: the mean AMPK and HIF-1 signatures for the indicated time points of each subclone. Error bars mean ± SEM.


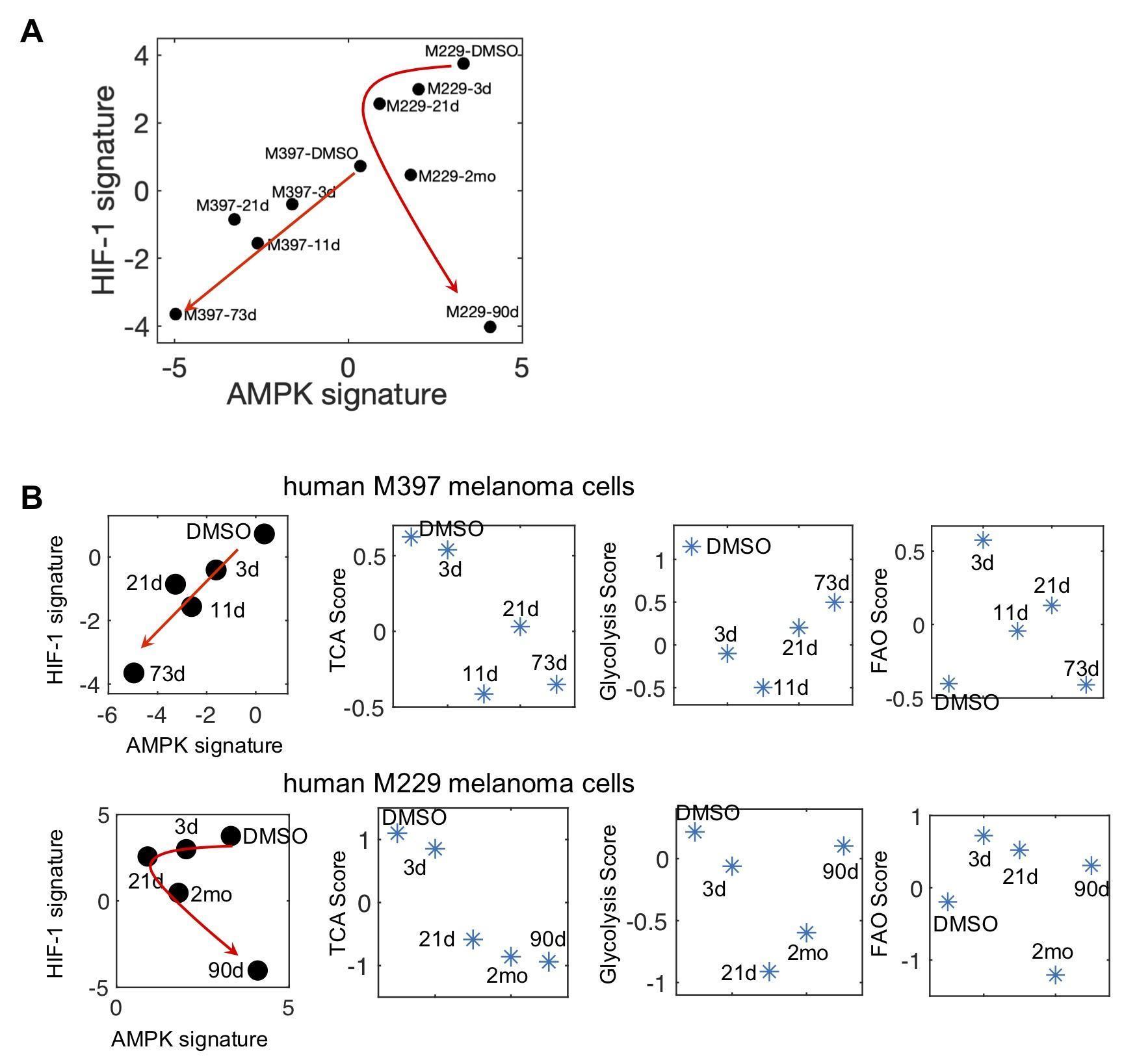


**Supplementary Figure 4 (FigS4)**: The human melanoma M397 and M229 cells exhibit L/L signature when cultured with vemurafenib. (A) The AMPK and HIF-1 signatures of M397 and M229 cells upon the treatment of DMSO or vemurafenib for the indicated time points. (B) The metabolic pathway scores of M397 and M229 cells upon the treatment. The red arrows indicate the change of AMPK and HIF-1 signatures upon the treatment. The RNA-seq data of M397 and M229 were obtained from Gene Expression Omnibus (GEO) with the series ID GSE110054.


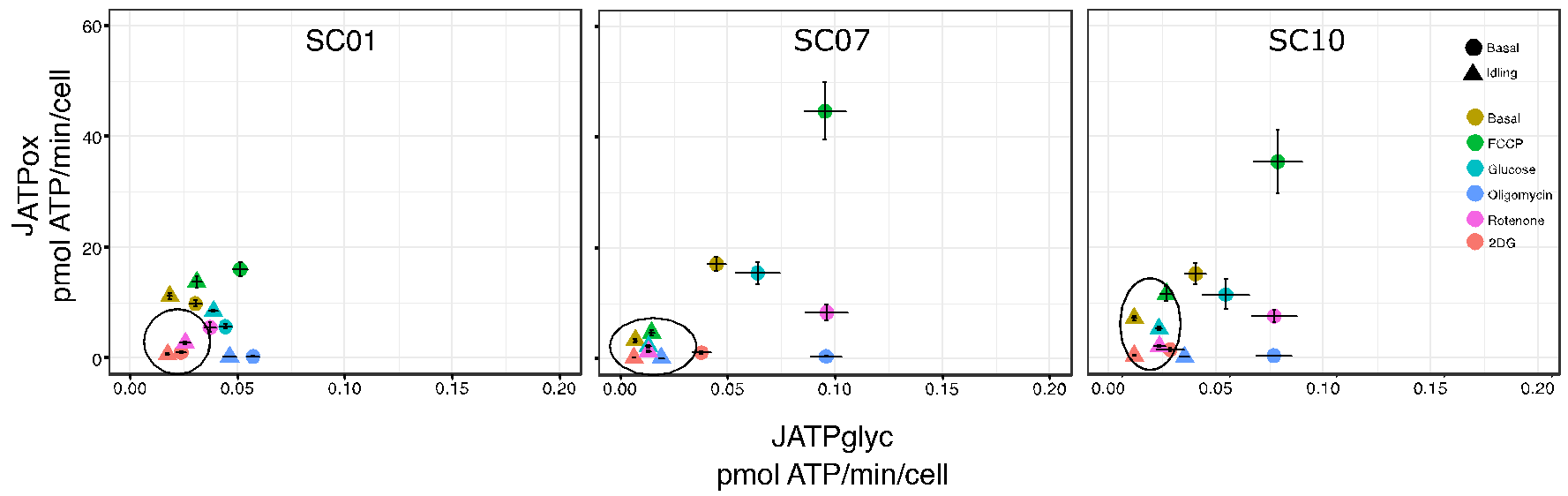


**Supplementary Figure 5 (FigS5)**: Quantification the ATP production (J_ATP_) from extracellular flux measurements. Oxygen consumption rates (OCR) from Seahorse Mito Stress Test, and Extracellular Acidification Rates (ECAR) from Seahorse Glyco Stress Test were used to calculate the total rate of ATP production via either oxidative phosphorylation (J_ATPox_ pmolATP/min/cell) or glycolysis (J_ATPglyc_ pmolATP/min/cell) for three single-cell derived SKMEL5 subclones, SC01, SC07, & SC10 in different metabolic substrates as indicated. Circle represents the ATP production in baseline, while the triangle represents the ATP production in the idling state induced by 8μM PLX4720.


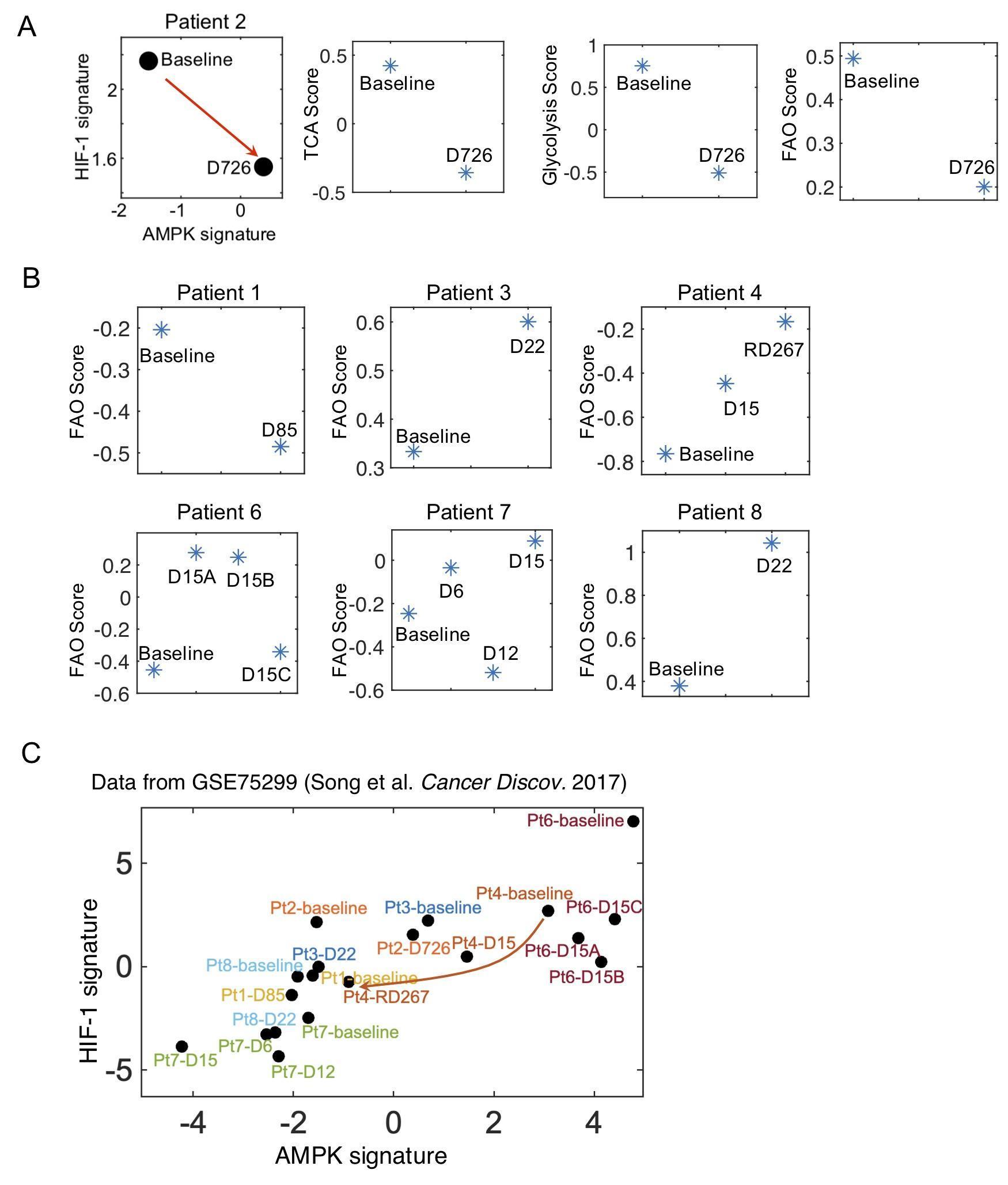


**Supplementary Figure 6 (FigS6)**: The melanoma patient samples exhibit L/L signature upon MAPKi treatment. (A) The AMPK/HIF-1 signatures and the metabolic pathway scores of the samples from patient 2 before and during treatment. The melanoma sample exhibits decreased HIF-1a activity but increased AMPK activity upon long-term MAPKi treatment. (B) The FAO scores of melanoma samples from patients 1, 3, 4, 6, 7 and 8 before and during the treatment. (C) The AMPK and HIF-1 signatures of all patient samples. The orange arrow indicates that the melanoma samples from patient 4 decrease both the AMPK and HIF-1 activities upon the treatment. The same change of AMPK and HIF-1 activity is also observed for patients 1, 3, 6, 7 and 8. The RNA-seq data of patient samples were obtained from GEO with the series ID GSE75299.


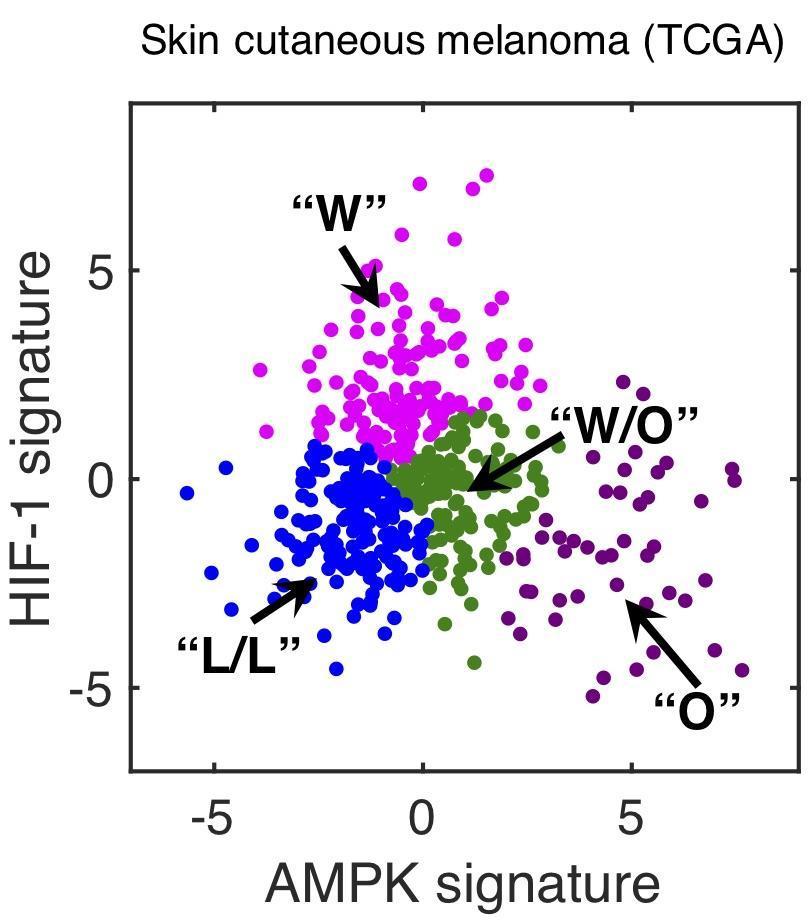


**Supplementary Figure 7 (FigS7)**: The skin cutaneous melanoma patient samples obtained from The Cancer Genome Atlas (TCGA) exhibit distinct metabolic states. Based on the AMPK/HIF-1 signatures, the patient samples can be grouped into four clusters corresponding to a glycolysis state (“W”), an OXPHOS state (“W/O”), a hybrid metabolic state (“W/O”) and a metabolically L/L state (“L/L”). Different colors represent different metabolic states. The RNA-seq data of 472 melanoma patient samples were obtained from TCGA at cBioPortal. k-means clustering analysis was applied to group the patient samples into 4 metabolic states.


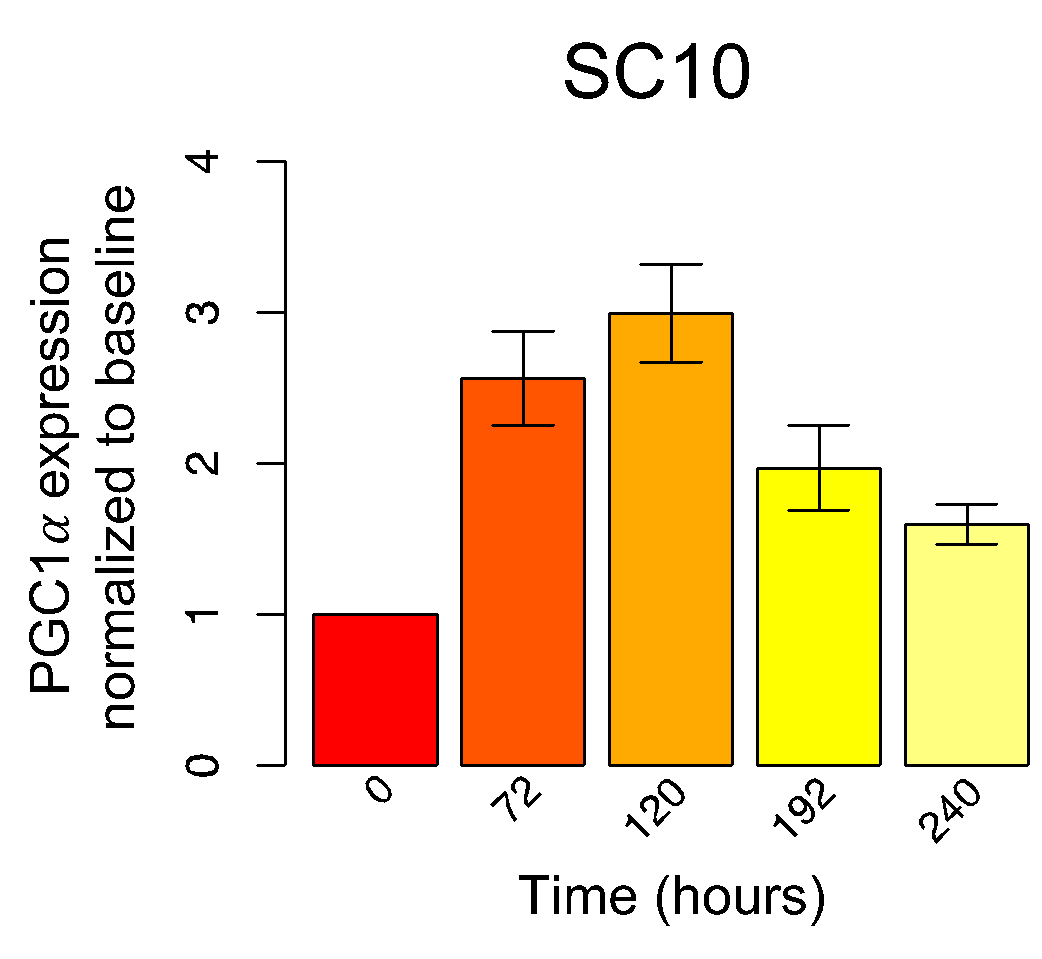


**Supplementary Figure 8 (FigS8)**: PGC1$\alpha$ activation upon BRAFi treatment is transient. Barplot showing the expression of mitochondrial biogenesis master regulator, PGC1$\alpha$, quantified by qPCR in the subclone SC10 in 8μM PLX4720 in the indicated times post treatment. The expression is normalized to the expression at time 0. Bar-plot shows $mean\pm SEM$ from 3+ replicates.


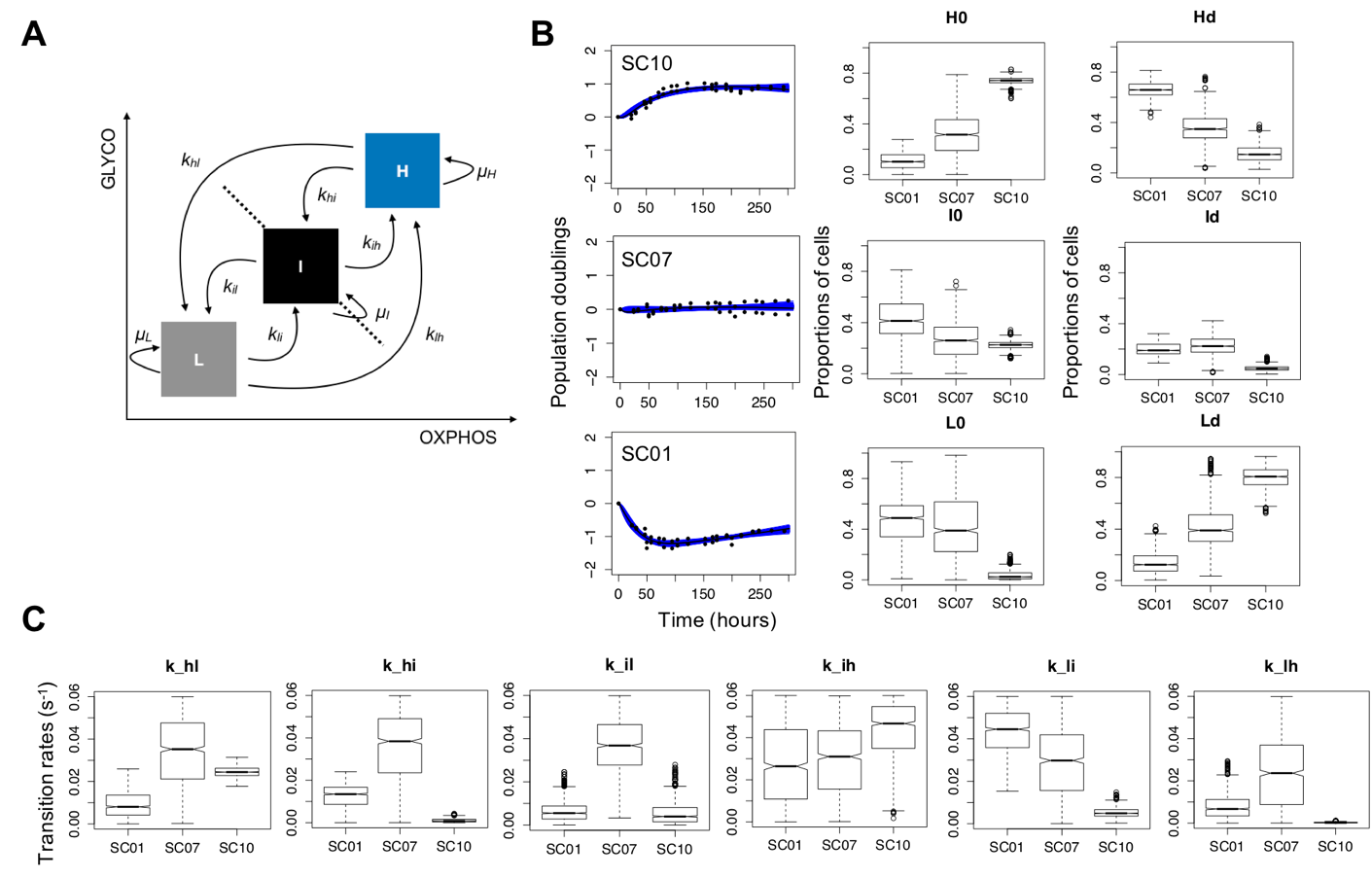


**Supplementary Figure 9 (FigS9)**: A three-state model of cell population qualitatively captures the differential drug responses of SC01, 07 and 10. (A) Schematic illustration of the three-state model in 2-dimensional (2D) metabolic space, defined in terms of OXPHOS and Glycolysis. Tumor cells can exist in three metabolic states, High-High (H), Low-Low (L), and High-Low or Low-High (grouped as intermediate or I). Each state has its unique drug-induced proliferation rate, and tumor cells can transition among states. (B) (*Left panel*) model can describe the BRAFi-induced responses of SC01, SC07, and SC10; dark circles represent the log2-normalized experimentally measured cell counts over time in 8$\mu$M PLX4720, while the blue curve represents the MCMC fitted model ensemble; (*Middle panel*) the model-predicted initial proportions of cells in each subclone in three metabolic states; (*Right panel*) the model-predicted drug-induced proportions of cells in three metabolic state in each subclone in the idling state. (C) Transition rates obtained from MCMC parameter calibration.

**Supplementary Figure 10 (FigS10)**: MCMC trace plots for three subclones. Shown are the total 5*10^4^ MCMC iterations, for the simulations the last 50% were used accounting for burn-in. Rate constants for: SC01, SC07, and SC10.


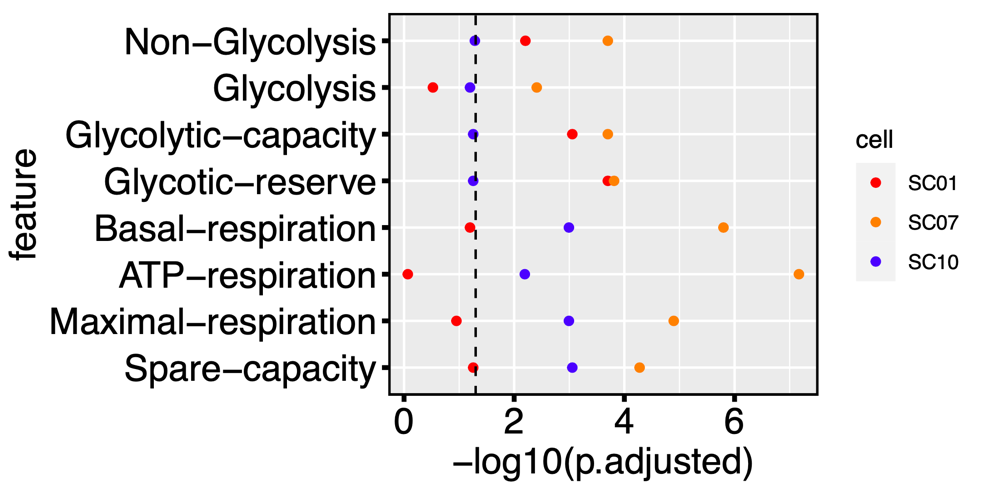


**Supplementary Figure 11 (FigS11) Statistical significance of the comparison results in figures 2D-E.** Seahorse metabolic features extracted from dynamic plots shown in Figure 2D were compared between each subclone at idling state vs baseline levels.

# **Supplementary References:**

1. [Jia D, Lu M, Jung KH, Park JH, Yu L, Onuchic JN, et al. Elucidating cancer metabolic plasticity by coupling gene regulation with metabolic pathways. Proc Natl Acad Sci U S A. 2019;116:3909–18.](http://paperpile.com/b/L3NbwQ/pBq00)

2. [Hardeman KN, Peng C, Paudel BB, Meyer CT, Luong T, Tyson DR, et al. Dependence On Glycolysis Sensitizes BRAF-mutated Melanomas For Increased Response To Targeted BRAF Inhibition. Sci Rep. 2017;7:42604.](http://paperpile.com/b/L3NbwQ/238dq)

3. [Paudel BB, Bishal Paudel B, Harris LA, Hardeman KN, Abugable AA, Hayford CE, et al. A Nonquiescent “Idling” Population State in Drug-Treated, BRAF-Mutated Melanoma [Internet]. Biophysical Journal. 2018. page 1499–511. Available from:](http://paperpile.com/b/L3NbwQ/eOec) <http://dx.doi.org/10.1016/j.bpj.2018.01.016>

4. [Zhou JX, Pisco AO, Qian H, Huang S. Nonequilibrium population dynamics of phenotype conversion of cancer cells. PLoS One. 2014;9:e110714.](http://paperpile.com/b/L3NbwQ/XSZx)

5. [Soetaert K, Petzoldt T, Woodrow Setzer R. Solving Differential Equations inR: PackagedeSolve [Internet]. Journal of Statistical Software. 2010. Available from:](http://paperpile.com/b/L3NbwQ/X3Yr) <http://dx.doi.org/10.18637/jss.v033.i09>

6. [Soetaert K, Petzoldt T. Inverse Modelling, Sensitivity and Monte Carlo Analysis inRUsing PackageFME [Internet]. Journal of Statistical Software. 2010. Available from:](http://paperpile.com/b/L3NbwQ/LyZi) <http://dx.doi.org/10.18637/jss.v033.i03>
